# Robotic Repair of Right-Sided Direct Inguinal, Indirect Inguinal, Femoral, and Obturator Hernias (Quadruple Groin Hernias)

**DOI:** 10.7759/cureus.111887

**Published:** 2026-07-01

**Authors:** John J Kessler, Stephen Kavic, Hugo J Bonatti

**Affiliations:** 1 Surgery, University of Maryland School of Medicine, Baltimore, USA

**Keywords:** inguinal hernia repair, inguinal hernia surgery, mesh inguinal hernioplasty, rare inguinal hernia, robotic-assisted inguinal hernia repair

## Abstract

Inguinal, femoral, and obturator hernias are a bulge or protrusion of abdominal contents into the inguinal canal, femoral ring, and obturator ring, respectively. Only a few cases of combined inguinal and femoral or obturator hernias have been reported. Laparoscopic and robotic repair seems safe and feasible in these cases. A 74-year-old male presented with significant pain and a bulge protruding in the right groin reaching into the scrotum. He consented to robotic-assisted inguinal hernia repair.

Eight mm trocars were placed into the umbilicus and to the left and right of it. On exploration, a right pantaloon hernia was visualized. The peritoneal flap was developed from a transverse incision above the hernia. On preperitoneal dissection, in addition, a femoral hernia was found. The contents of all three hernias were reduced, and the sack of the indirect hernia was degloved after the chord was dissected off. The lateral channel was developed in the usual fashion. Whilst developing the medial channel to below the pubic bone, an obturator hernia was found; the contents were reduced. The large medial inguinal hernia defect was closed with a running 3-0 barbed suture. The landing zone was fully developed. A large self-fixating mesh was placed covering all four hernia defects with appropriate overlap in all directions. The patient was discharged within three hours after surgery and had an uneventful recovery.

To the best of our knowledge, this is the first “quadruple” groin hernia repair using the robotic approach. During dissection in laparoscopic/robotic inguinal hernia repair, surgeons should actively look for additional defects.

## Introduction

A quadruple hernia is defined as four distinct hernias with the same laterality present concurrently in one patient. Inguinal hernias are a bulge or protrusion of abdominal contents into the inguinal canal. A direct inguinal hernia enters the inguinal canal medial to the inferior epigastric vessels via Hesselbach’s triangle, while an indirect inguinal hernia enters the inguinal canal lateral to the inferior epigastric vessels via the internal inguinal ring. They constitute the vast majority of groin hernias, with indirect inguinal hernias accounting for approximately 50% and direct inguinal hernias comprising 30-40% of cases. A pantaloon hernia is defined as concomitant indirect and direct inguinal hernias. Femoral hernias, a bulge or protrusion of abdominal contents through the femoral ring, follow as the next most common subtype (<10%), while obturator hernias, a bulge or protrusion of abdominal contents through the obturator canal, are rare (<1%) [1-3]. Globally, groin hernias represent a significant public health burden, with over 32 million patients diagnosed with an inguinal hernia and more than 20 million undergoing groin hernia surgery annually [3,4]. These figures underscore the high prevalence of groin hernias as one of the most common surgical conditions worldwide, particularly among men.

The clinical management of groin hernias is further complicated by the occurrence of multiple defects. Multiple groin hernias are encountered in up to 23% of patients, with 6% demonstrating multiple ipsilateral groin hernias [4]. Such synchronous hernias pose diagnostic and therapeutic challenges, as they increase the risk of missed defects during surgery, potentially leading to recurrence or persistent symptoms. Combined ipsilateral inguinal and femoral hernias, or inguinal and obturator hernias, are infrequently reported in the literature, with only a handful of documented cases [5-9]. Even rarer is the presence of three concurrent groin hernias; one previous case report described a patient with three groin hernias occurring bilaterally [10].

A comprehensive literature search was conducted in PubMed from inception through 2025. The search strategy utilized a combination of Medical Subject Headings (MeSH) and free-text terms related to "multiple groin hernia," "inguinal hernia", "femoral hernia," and obturator hernia". The results were limited to peer-reviewed articles published in the English language. Our search did not identify any simultaneous occurrence of all four major types of groin hernias: indirect inguinal, direct inguinal, femoral, and obturator, on the same side. In this report, we present a unique case of four synchronous ipsilateral groin hernias involving an indirect inguinal hernia, direct inguinal hernia, femoral hernia, and obturator hernia. This rare anatomical presentation highlights the importance of thorough preoperative imaging and meticulous intraoperative exploration in patients with complex groin hernia disease. By documenting this case, we aim to raise awareness among surgeons and radiologists of the potential for multiple coexisting groin defects and contribute to the limited body of literature on such exceptional presentations. 

This article was previously presented as a meeting abstract at the 142nd German Surgery Congress on March 20, 2025. 

## Case presentation

A 74-year-old male presented to the clinic with a painful bulge in his right groin. The bulge had been present for six months and had begun to cause discomfort intermittently over five days. He had no comorbidities and no history of prior groin surgery or groin hernia. On exam, the bulge was non-tender, not reducible, and protruding through his right inguinal canal. As a result, he was diagnosed with a right incarcerated inguinal hernia. No preoperative imaging was obtained. The patient was admitted the same day and provided consent for a robotic-assisted right laparoscopic trans abdominal preperitoneal (TAPP) inguinal hernia repair.

**Figure 1:**
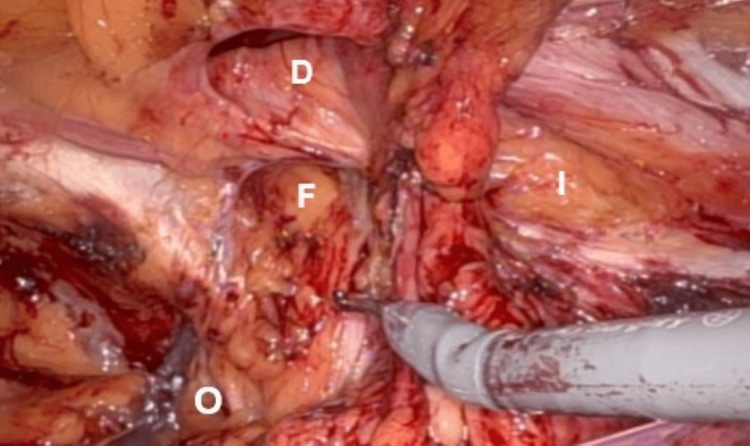
Operative view of four ipsilateral concurrent groin hernias D: direct inguinal hernia, I: indirect inguinal hernia, F: femoral hernia, O: obturator hernia

Abdominal entry with a 5mm Fios optical trocar at Palmer's point allowed for visualization of the peritoneal cavity. Two 8mm trocars were placed in the right mid-abdomen and left of the umbilicus. The 5 mm port was exchanged for an 8 mm port. Adhesions between the small bowel and the abdominal wall from prior abdominal surgery were taken down. On exploration, a right pantaloon hernia was immediately visualized. The peritoneal flap was developed from a transverse incision above the hernia. On preperitoneal dissection, a right femoral hernia was incidentally identified and reduced. The contents of all three hernias (indirect, direct, and femoral) were reduced. All bowel was observed to be viable post-reduction. Next, the sack of the indirect hernia was degloved after the cord was dissected off. The lateral channel was developed in the usual fashion. Whilst developing the medial channel to below the pubic bone, an obturator hernia was identified, and the contents were reduced (Figure 1). The large medial inguinal hernia defect was closed with a running 3-0 barbed suture in two layers. The lateral defect was also closed with a running 3-0 barbed suture in two layers. Subsequently, the landing zone was fully developed. A large self-fixating mesh was placed covering all four hernia defects with appropriate overlap in all directions, with special attention to the inferior medial aspect below the pubic bone for obturator hernia coverage. The peritoneal flap was sutured to cover the mesh. A redundant hernia sack was used to cover a defect in the peritoneal flap. The operative video is shown in Video 1. The patient was discharged within three hours after surgery and had an uneventful recovery. No hernia recurrence has been identified at the six-month follow-up.

**Video 1:** Operative video

## Discussion

To the best of our knowledge, this is the first “quadruple” ipsilateral groin hernia identified and repaired using the robotic approach. We report this case because of the novelty and potential impact on surgical complications. Occult obturator and femoral hernias are rarely identified on physical exams, especially when an inguinal hernia is present. Unfortunately, obturator hernias have increased rates of morbidity and mortality compared to inguinal and femoral hernias. The morbidity rates of obturator hernias are 38%, and the mortality rate is 13-40%, with an increase up to 70% in strangulated obturator hernias, while inguinal hernias, when strangulated, have morbidity and mortality rates of 27% and 3-5%, respectively [3,4].

Recurrence is one of the most feared complications of all hernia surgeries and occurs in 1-2% of groin hernia repairs with mesh [11]. While an increase in the rate of recurrence has not been identified in patients with multiple ipsilateral groin hernias compared to those with a single groin hernia, occult hernias have been seen at increased rates in recurrent inguinal hernia repair. Ipsilateral concurrent groin hernia rates are identified in 6% of patients on initial repair, and the rate of identification of these occult hernias increases to 9-38% upon reoperation for recurrence [12]. Additionally, 38.1% of patients with a new diagnosis of femoral hernia previously had an ipsilateral inguinal hernia repair [12]. One possible explanation for these findings is that these femoral hernias were present but overlooked during the original operation, and reoperation allowed for the identification of these occult hernias.

Minimally invasive techniques have become widely used in groin hernia repair. TAPP and totally extraperitoneal (TEP) can be performed either laparoscopically or with robotic assistance. Once proficiency has been reached, these minimally invasive surgical approaches have been proven equivalent to open repair regarding complications and recurrence [13,14]. One study found that laparoscopic TEP hernia repair was superior to open surgery in its ability to identify ipsilateral occult hernias; however, other studies have not supported this finding [15,16]. One limitation of TAPP repair is that hernias not containing peritoneal structures are less likely to be identified unless the peritoneal flap is extended to allow visualization of the less common hernia locations [17]. During dissection in laparoscopic/robotic inguinal hernia repair, surgeons should actively look for additional defects on the ipsilateral side of the known defect. Full dissection of the myopectineal orifice allows for identification of incidental ipsilateral hernias. These incidental findings should be repaired during the initial operation with prior appropriate informed consent. Incorporating ipsilateral occult hernia repair into the originally planned mesh repair allows for easy dissection in a virgin surgical field and prevents future progression and development of complications that could require reoperation. Additionally, ipsilateral multiple hernia repairs do not increase operative time, rates of complications, or length of stay [4].

## Conclusions

A “quadruple hernia” consisting of ipsilateral indirect inguinal, direct inguinal, femoral, and obturator hernias has now been identified in a single patient. To the best of our knowledge, this represents the first reported case of all four major groin hernia types occurring synchronously on the same side. This exceptional anatomical presentation underscores the remarkable variability of groin anatomy and the potential for multiple coexisting defects that may not be apparent on routine clinical examination. This case highlights several important clinical lessons for surgeons managing groin hernias. First, it reinforces the consideration for preoperative imaging in patients with complex or recurrent groin symptoms to better delineate all possible hernia defects. Second, during minimally invasive (laparoscopic or robotic) groin hernia repair, surgeons should maintain a high index of suspicion and consider a systematic, complete exploration of the entire myopectineal orifice, including the inguinal, femoral, and obturator regions. Failure to identify and address all defects intraoperatively can result in missed hernias, persistent symptoms, or early recurrence.

In conclusion, the presence of multiple synchronous groin hernias, although uncommon, is likely under-recognized. Routine vigilance for additional defects should become standard practice in groin hernia surgery, especially in minimally invasive approaches where excellent visualization of the entire groin anatomy is possible. Reporting rare cases such as this contributes to the surgical literature and may improve awareness, diagnostic accuracy, and long-term outcomes for patients with complex groin hernia disease.
